# Five Decades Later, Are Mesenchymal Stem Cells Still Relevant?

**DOI:** 10.3389/fbioe.2020.00148

**Published:** 2020-02-28

**Authors:** Mario Gomez-Salazar, Zaniah N. Gonzalez-Galofre, Joan Casamitjana, Mihaela Crisan, Aaron W. James, Bruno Péault

**Affiliations:** ^1^MRC Centre for Regenerative Medicine and Centre for Cardiovascular Science, The University of Edinburgh, Edinburgh, United Kingdom; ^2^Orthopaedic Hospital Research Center and Broad Stem Cell Research Center, David Geffen School of Medicine, University of California, Los Angeles, Los Angeles, CA, United States; ^3^Department of Pathology, Johns Hopkins University, Baltimore, MD, United States

**Keywords:** tissue engineering, mesenchymal stem cell, pericyte, cell therapy, adventitia

## Abstract

Mesenchymal stem cells are culture-derived mesodermal progenitors isolatable from all vascularized tissues. In spite of multiple fundamental, pre-clinical and clinical studies, the native identity and role in tissue repair of MSCs have long remained elusive, with MSC selection *in vitro* from total cell suspensions essentially unchanged as a mere primary culture for half a century. Recent investigations have helped understand the tissue origin of these progenitor cells, and uncover alternative effects of MSCs on tissue healing *via* growth factor secretion and interaction with the immune system. In this review, we describe current trends in MSC biology and discuss how these may improve the use of these therapeutic cells in tissue engineering and regenerative medicine.

## Introduction

Three main classes of stem cells can be used, in theory, for tissue regeneration and engineering. Organ resident, lineage specific stem cells should be ideal candidates but are rare, difficult to identify and purify and usually impossible to “expand” in culture. Conversely, culture adapted pluripotent stem cells may exhibit ultimate therapeutic potential, should their engraftment, differentiation and cycling be accurately controlled (reviewed in Robinton and Daley, 2012). In a third category fall multipotent cells endowed with mesodermal differentiation potential that proliferate in extended cultures of dissociated pre- and post-natal vascularized tissues, the prototype of which is the mesenchymal stem cell. MSCs have been used in around 1000 clinical trials (see ClinicalTrials.gov) in multiple indications as diverse as musculo-skeletal defects, disorders of the immune system including auto-immune diseases, and myocardial infarcts. In spite of this popularity, the MSC remains a biologic enigma, since retrospective derivation in culture has long concealed the true native identity of this cell, the role of which in tissue regeneration is also incompletely understood. Initially defined as a true stem cell driving cell-for-cell replacement, the MSC is now recognized primarily as a growth factor secretor and immunomodulatory agent (Sacchetti et al., 2007; Caplan, 2017). These combined functions drive tissue healing and rejuvenation, although their respective contributions to tissue repair remain unknown.

We have herein collated classic and recent results on mesenchymal stem cell phenotype, potentials and innate identity, and speculated about the future of MSCs in cell therapies and tissue engineering.

## Historical Perspective

From the mid 1960’s, the soviet scientist Alexander Friedenstein demonstrated that mouse bone marrow and other blood-forming organs contain clonogenic progenitor cells that can give rise in culture to fibroblasts, as well as other mesodermal cells (Friedenstein et al., 1966, 1970, 1974, 1987). He observed that these cells do not belong to the hematopoietic cell lineage and have the ability to give rise to bone and cartilage-forming cells.

Friedenstein’s studies were pursued by Owen and Friedenstein (1988) and Piersma et al. (1985). These, and further investigations (Friedenstein et al., 1987; Wakitani et al., 2002) established that such cells isolated by plastic adherence can form osteoblasts, chondrocytes, adipocytes and myoblasts. Hence, multipotent progenitors cultured from total mouse bone marrow were shown to exhibit developmental plasticity, giving rise to diverse mesodermal cell lineages. These cells would subsequently be termed “mesenchymal stem cells” by Arnold Caplan, who drew a parallel with the stem cells at the origin of mesodermal tissues in the embryo, and was also the first one to grow these cells from human tissues (Caplan, 1991).

Haynesworth et al. (1992b) cultured and expanded bone marrow MSCs from the iliac crest of human donors. Culture adherent cells were subcultured and tested for their potential to differentiate into cartilage and bone *in vivo*, finally showing that the human bone marrow also contains cells with osteogenic potential that can be grown in culture (Haynesworth et al., 1992b). Of important note, MSCs were found to produce fibrocartilage, and not the hyaline cartilage that lines articular surfaces in joints, representing the target regenerative cell for the treatment of osteoarthritis. Identifying strategies and tactics to solve this major shortcoming of MSC-related chondroprogenitors remains, almost 20 years later, the object of intense research (Anderson et al., 2018). The same group generated monoclonal antibodies identifying SH-2 and SH-3 as unique cell surface antigens on MSCs (Haynesworth et al., 1992a). A few years later (Barry et al., 1999, 2001) described the ligands of the SH-2 and SH-3 antibodies as CD105 and CD73, respectively. From this point, MSCs could be selected on 1- ability to adhere and proliferate in culture, 2- expression of cell surface markers: CD73, CD90, CD105, CD44, CD124 (Haynesworth et al., 1992a; Barry et al., 1999, 2001), and 3- capacity to give rise to mesodermal cell lineages *in vitro*. MSCs express bone cell markers such as alkaline phosphatase, and when induced under specific conditions form mineralizing colonies with increased expression of other bone differentiation markers (Simmons and Torok-Storb, 1991; Gronthos et al., 1994; Waller et al., 1995).

Pittenger et al. (1999) isolated presumptive MSCs expressing neither the lipopolysaccharide receptor CD14, nor CD34 and the hematopoietic cell marker CD45 from marrow aspirates from multiple donors between 19 and 57 years of age. MSCs from over 50 donors were expanded, all responding positively to osteogenic, adipogenic and chondrogenic inductions. No spontaneous differentiation was observed during expansion, and the cells displayed normal karyotype and telomerase activity until passage 12 (Pittenger et al., 1999). In addition, mesenchymal stem cells provide limited *in vitro* support to hematopoietic stem cells (Majumdar et al., 1998), and favor tendon regeneration in the rabbit (Young et al., 1998).

Although bone marrow was the first organ to be studied as a source of MSCs, cells isolated from adult adipose tissue, which remains a major provider of MSCs, demonstrated similar multipotency *ex vivo* (Zuk et al., 2002; Rodriguez et al., 2005; Xu et al., 2005; Rodeheffer et al., 2008). These findings were extended to multiple other organs, concluding that most – if not all – vascularized tissues contain presumptive MSCs (Gronthos et al., 2000; Arai et al., 2002; Romanov et al., 2003; Mansilla et al., 2006; Zheng et al., 2007; Crisan et al., 2008).

Because of increasing interest in MSCs and growing clinical relevance thereof, a need to establish a non-ambiguous and broadly accepted definition for these cells arose. The International Society for Cellular Therapy proposed four minimum criteria to define an MSC for research purposes (Dominici et al., 2006):

• Be plastic adherent
• Express the cell surface antigens CD105, CD90, and CD73
• Not express the cell surface antigens CD45, CD19, CD14, CD11b, CD34, CD79α, and HLA-DR
• Have the capacity to differentiate into osteoblasts, chondrocytes and adipocytes

It is essential to remember that these biologic characteristics are used to identify cultured MSCs in the laboratory, and represent by no means sufficient and accepted release criteria for stocks of MSCs to be used therapeutically in patients.

## A Note on Cell Nomenclature: What’s in an Acronym?

**FIGURE 1:**
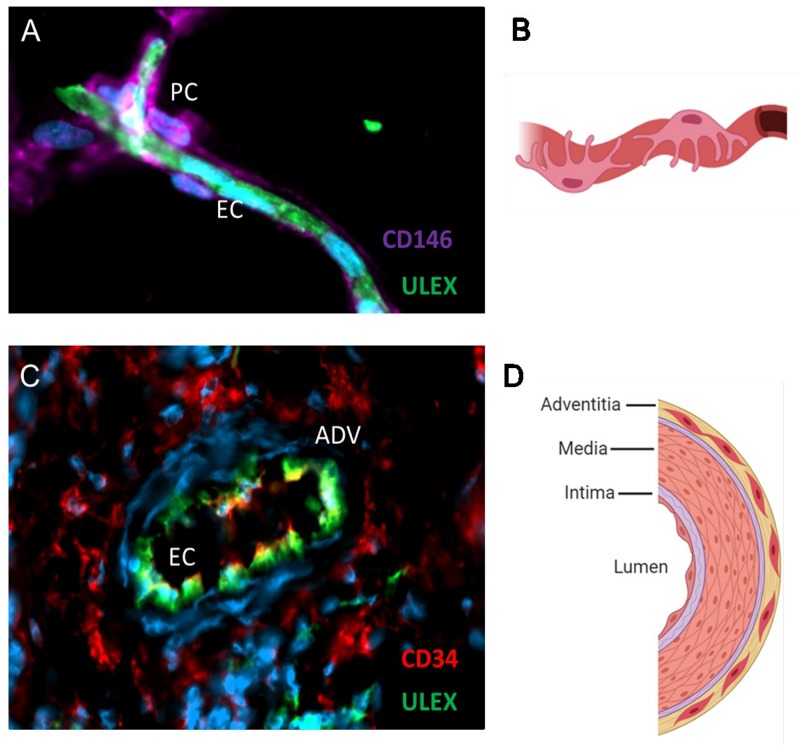
MSC progenitors are located in capillaries and large vessels. Immunofluorescence analysis of adipose tissue **(A)** and schematic **(B)** showing pericytes expressing CD146 in close contact with the endothelium stained with the Ulex europaeus lectin. Blue marks DAPI staining of cell nuclei. Adventitial cells expressing CD34 are located in the adventitial layer of veins and arteries **(C,D)**. Endothelial cells appear yellow/green because they express both CD34 and the Ulex receptor. Schematics were created with Biorender.com.

Mesenchymal stem cells have been frequently re-baptized. While some new appellations, such as “mesenchymal progenitor cells,” “multipotent adult stem cells” (Beltrami et al., 2007) or “multipotent adult progenitor cells” (Jiang et al., 2002) diverged only slightly from the original concept, others, like “mesenchymal stromal cells” or “multipotential stromal cells,” although respecting the MSC acronym, introduced a radical difference in terms of biologic significance (Zimmermann et al., 2003). Even though MSCs exhibit some attributes of stem cells: multipotency within the mesodermal cell lineage and some self-renewal in culture, they do not meet the full criteria for qualification as *bona fide* stem cells, notably with respect to permanent cell lineage repletion *in vivo*, and a different name is needed, but why “stromal?” Stromal cells constitute the supporting architecture of an organ, and are distinct from the cell compartments involved in organ function. Juxtaposition of these antithetical terms in the commonly used “mesenchymal stem/stromal cell” adds to the confusion. Did the fibroblastic appearance of MSCs suggest the use of the adjective “stromal?” Did adoptively transferred MSCs ever contribute stromal cell populations in the host? The lexical justification of MS(tromal)Cs, that were born at about the same time, and in the same research group (Lazarus et al., 1995), as MS(tem)Cs, is, consequently, not clear. However, much more recent work that identified MSC natural forerunners as pericytes, perivascular fibroblasts and adventitial cells (Figure 1) (Crisan et al., 2008; Corselli et al., 2012) may have confirmed a stromal origin for MSCs, with the caveat that MSCs are profoundly modified by *in vitro* culture (see below) and probably retain little memory of their perivascular ancestors. In the latest episode of MSC renaming, and to convey the notion that these cells function in tissue repair primarily by releasing growth factors and cytokines, Arnold Caplan, who initially coined the term “mesenchymal stem cell,” proposed to replace it by “medicinal signaling cells” (Caplan, 2017). For the sake of simplicity though, and optimal bibliographic accessibility through keyword searches, we have used “mesenchymal stem cell” uniformly in the present article, although this is more reflective of tradition than scientific accuracy.

## *In Situ* Counterparts of Cultured MSCs

Historically, MSCs were isolated in culture, being selected on the ability of a cell subset(s) to adhere and proliferate for several weeks of primary cultivation. For decades MSCs were thus retrospectively isolated cells of unknown original identity, tissue distribution, frequency, and natural function *in vivo*. Typically, the MSC description provided by ISCT in 2006 – that is, about 40 years after Friedenstein’s original observations – still relied largely on markers defined in culture (Dominici et al., 2006), giving no clue as to the innate nature of these cells *in situ.* However, from about the same time, phenotypic correlations started suggesting a native perivascular localization for MSC like progenitor cells in humans (Schwab and Gargett, 2007; Traktuev et al., 2008) and mice (Brachvogel et al., 2005; Sacchetti et al., 2007). In a large-scale study of multiple human tissues, some of us identified vascular pericytes by immunohistochemistry, then purified those to homogeneity by flow cytometry. Cultured pericytes, notwithstanding the tissue of origin, were indistinguishable from conventional MSCs in terms of adherence to plastic, morphology, phenotype, proliferation rate, and developmental potential (Crisan et al., 2008). Importantly, the same study documented native expression by human pericytes of the canonical MSC markers CD73, CD90, and CD105, further supporting the hypothesis that both cell types are affiliated. Altogether, these results designated microvascular pericytes as at least one class of tissue resident MSCs (Crisan et al., 2008), even though it was not known whether these perivascular cells *in situ* could be also functionally qualified as mesenchymal stem cells, or were only the precursors thereof. Pericytes are not the only perivascular cells endowed with the potential to give rise to MSCs, which are therefore not necessarily associated natively with capillaries and microvessels. A population of fibroblast like progenitors located in the outermost layer of larger arteries and veins, the *tunica adventitia*, was also identified as a source of *bona fide* MSCs. Adventitial progenitors are phenotypically and anatomically distinct from pericytes. However, like pericytes, adventitial cells natively express MSC markers and give rise to MSCs in culture (Corselli et al., 2012). In this regard, pericytes and adventitial cells have been collectively termed perivascular stem cells (PSCs).

## Mesenchymal Progenitors in the Native “Niche”: Organ Specializations

Presumptive MSCs are found notably among pericytes and adventitial cells in the perivascular niche, and possibly as interstitial fibroblast like cells in other territories. The transcriptome and phenotype of perivascular cells is profoundly modified during *in vitro* expansion, hence resulting MSCs are very different from their native, tissue resident ancestors (Hardy et al., 2017; Gomez-Salazar et al., in preparation). Whether cells identical to cultured MSCs exist and function *in vivo* is not known. Perivascular pre-MSCs have been characterized in multiple tissues. Since blood vessels, hence perivascular cells, are present in virtually all organs, an important question is whether perivascular cells from different anatomic locations differ in terms of stem cell activity and support.

In the bone marrow, pericytes have been characterized as both supporting and repressing haematopoietic stem cell (HSC) activity. Distinct subpopulations of pericytes identified with surface markers play regulatory roles in HSC homing, maintenance, and quiescence, mostly via chemokine secretion. Most relevant subpopulations include CXCL12 + reticular cells (Sugiyama et al., 2006; Corselli et al., 2013), and nestin- (Méndez-Ferrer et al., 2010; Ding et al., 2012), NG2- (Kunisaki et al., 2013), CD146- (Sacchetti et al., 2007; Corselli et al., 2013; Isern et al., 2013) and leptin receptor- (Ding et al., 2012; Ding and Morrison, 2013) positive cells (reviewed by Sá da Bandeira et al., 2017). However, although these observations point to the existence of discrete subsets of hematopoiesis supporting perivascular cells in blood-forming tissues, the organ distribution of these stromal cells was found to be amazingly non-specific, since pericytes sorted from human skeletal muscle and adipose tissue robustly and lengthily support hematopoietic progenitor cells in culture (Corselli et al., 2013).

In the human kidney, pericytes surrounding juxtaglomerular arterioles, as well as their MSC progeny in culture, produce renin, an enzyme responsible for the production of angiotensin I, a regulator of blood pressure, and this secretory potential is sustained by their cultured MSC progeny (Stefanska et al., 2016; Shaw et al., 2018). Within the intestine, CD34+ mesenchymal cells are an important component of the stem cell compartment that maintains intestinal epithelial stem cells at homeostasis and is activated after intestinal injury (Stzepourginski et al., 2017).

## Native Perivascular Progenitor Cells and Derived MSCs Are Antigenically and Functionally Heterogeneous

MSCs have long retained a “one-cell-does-it-all” image, where cells cultured indifferently from the bone marrow or subcutaneous fat along a unique protocol can be used to treat conditions as diverse as auto-immune diseases, bone fractures or limb ischaemia. MSCs are heterogenous, as can be expected from cultures of unseparated, total cell suspensions, although this complexity becomes reduced over time *in vitro*, possibly allowing better protocol standardization. Accordingly, clonal analysis of extended MSC cultures has shown that diversity is dramatically lowered to just a few clones after multiple passages (Selich et al., 2016). Moreover, MSC clones exhibit diverse differentiation potentials, only one third developing into the three canonical mesodermal cell lineages (Muraglia et al., 2000; Hardy et al., in review). Whether such MSC clonal selection depends on the organ source, demographics of the donor, tissue processing and culture conditions, and affects the therapeutic performance of the cells is unknown but thorough analysis of these variables should guide protocol definition.

The heterogeneity of conventional, culture derived MSCs may also reflect the intrinsic diversity of their native forerunners. Direct analysis of perivascular presumptive MSCs has revealed that, within a given tissue or organ, these cells are phenotypically and functionally diverse. A developmental hierarchy of pericytes and adventitial perivascular cells has been established in human adipose tissue by single-cell transcriptome analysis (Hardy et al., 2017). Correlatively, these two cell types which both contribute to conventional cultured MSCs play distinct roles in osteogenesis *in vivo* (Wang et al., 2019). Capoccia et al. (2009) found that bone marrow MSC like cells with high aldehyde dehydrogenase (ALDH) activity sustain better improvement of the ischaemic hind limb, as compared to the whole stromal cell population. It was recently confirmed that ALDH^hi^ perivascular progenitors are developmentally more primitive than their ALDH^lo^ counterparts (Hardy et al., 2017; Gomez-Salazar et al., in preparation). Perivascular cells and derived MSCs with superior chondrogenic potential have been identified by marker expression (Dickinson et al., 2017) and proximity to the joint (Hindle et al., 2016), while the subset of perivascular adventitial cells that express CD10 is considerably enriched in osteogenic progenitors, at the expense of adipogenic cells (Ding et al., 2019).

On the pathology side, sub-populations of perivascular cells have also been reported to contribute to fibrosis and vascular calcification. Upon injury, perivascular MSC progenitors drive the critical remodeling of the affected organ, reducing its function dramatically. Resident Gli1+ perivascular cells give rise to myofibroblasts upon renal, pulmonary, hepatic, or cardiac injury, contributing to organ failure, which is rescuable upon ablation of these cells (Kramann et al., 2015). Perivascular progenitors also contribute to vessel calcification (Leszczynska et al., 2016), as a cell subset marked by Gli-1 expression (Baker and Peault, 2016; Kramann et al., 2016), and in the bone marrow fibrosis can be targeted pharmacologically with the Gli1 inhibitor GANT61 (Schneider et al., 2017). Similarly, αv integrins on perivascular and interstitial cells in the skeletal and cardiac muscle contribute to fibrosis via TGFβ signaling after injury, that can be genetically controlled by αv integrin ablation, or pharmacologically alleviated by targeting αv integrins with small molecules (Murray et al., 2017). Furthermore, a subset of PDGFRβ+ perivascular cells co-expressing PDGFRα is highly fibrotic and contributes to fatty degeneration following massive tears of the mouse rotator cuff (Jensen et al., 2018).

## MSC Therapy: Immunomodulation and Other Actions Contributing to Injured Tissue Regeneration

MSCs have been used in clinical trials for almost two decades, since bone marrow cells were injected into patients undergoing high-dose chemotherapy for breast cancer (Koç et al., 2000). It is now well documented that MSCs release growth factors and cytokines along with extracellular vesicles to activate cell proliferation, prevent apoptosis, and ultimately improve regenerative responses. MSCs also modulate immune responses by decreasing inflammation and preventing scar formation. MSCs are able to suppress both CD4+ T helper cells and CD8+ cytotoxic T cells, inhibit activation of dendritic cells (DCs) and natural killer (NK) cells (Shi et al., 2012). Mechanisms by which MSCs prevent inflammation and promote healing are still not completely understood though. The immune modulatory effect of MSCs is mediated by both the release of soluble factors and direct contact with immune cells. The immunomodulatory capacity of MSCs by cell contact has been studied in depth, showing there is not a unique mechanism involved.

### MSC Immunomodulation by Cell-Cell Contact

Interaction of MSCs with T cells, dendritic cells, and natural killer cells requires the engagement of PD-1 (programmed death 1) with its ligands PD-L1 and PD-L2 for proper inhibition of proliferation and subsequent signaling by cytokines (Augello et al., 2005). The release of antibodies and other co-stimulatory molecules by B cells is also reduced after MSC administration. However, MSC-B cell interactions are not well understood and probably require both direct cell contact and indirect participation of other immune cells acting as intermediates (Fan et al., 2016). Indeed, CD3+ T cells are required for B-cell inhibition, otherwise the inhibitory effect of MSCs disappears and B cells proliferate (Rosado et al., 2015).

Likely reasons why MSCs do not activate the immune system is their lack of expression of the co-stimulatory molecules CD80 and CD86 (required for proper immune activation), absence of major histocompatibility complex (MHC) class II antigens (Le Blanc et al., 2003), and low expression of MHC class I molecules (Krampera et al., 2003; Nauta and Fibbe, 2007). MHC class I expression on MSCs seems to be particularly important to protect against NK cells, since MSCs affect NK cell cytotoxicity, likely by suppressing IL-2 induced cell activation (Spaggiari et al., 2006). Other molecules involved in cell-cell mediated immunomodulation are vascular cell adhesion protein 1 (VCAM1) (Ren et al., 2010) and galectin-1 (Gieseke et al., 2010). Lastly, galectin-9 is expressed by MSCs after activation with interferon-gamma (INF-γ) and seems to be a major mediator of proliferation, hence a marker of immunomodulatory potential (Ungerer et al., 2013).

However, notwithstanding the immunosuppressive effect of MSCs, it was recently shown that MSC apoptosis induced by CD8 + T cells may confer clinical benefits, and that complete lack of activation of the recipient host immune system was a predictor of clinical inefficiency. Apoptosis is crucial to the anti-inflammatory and regenerative activities of MSCs. In agreement, inducing apoptosis prior to MSC administration enhanced their efficacy (Galleu et al., 2017).

### MSC Immunomodulation by Soluble Factors

Soluble factors are also required for proper MSC-driven immune modulation. Indoleamine-pyrrole 2,3-dioxygenase (IDO), prostaglandin E2 (PGE2) and cyclooxygenase 2 (COX-2) are the main mediators of the immunosuppressive activity of MSCs in the presence of pro-inflammatory cytokines (Krampera et al., 2006; Ryan et al., 2007). PGE2 has been especially involved in the production of IL-10 by macrophages (Nemeth et al., 2009) and blocking differentiation of monocytes into dendritic cells (DCs) (Spaggiari et al., 2009). Interestingly, MSCs seem to be better immune cell modulators in the presence of IFN-γ and tumor necrosis factor alpha (TNF-α) by enhancing the production of PGE2 (Krampera et al., 2006; Ren et al., 2008). Indeed, there seems to be a correlation between activation of the immune system and the outcome of the treatment. For example, T cells from IFN-γ^–/–^ mice do not respond to MSCs, whereas MSCs pre-conditioned with IFN-γ are more efficient at suppressing graft vs. host disease (GvHD) (Polchert et al., 2008). This highlights the importance of immune activation as an indication of MSC treatment response. In addition to the factors mentioned above, other important molecules shown to be critical for immune modulation by MSCs are transforming growth factor-β1 (TGF-β1) (Nemeth et al., 2010), nitric oxide (NO), hepatocyte growth factor (HGF), IL-6 (Ghannam et al., 2010) and leukemia inhibitory factor (LIF) (Najar et al., 2010).

### MSCs in Tissue Regeneration: Cell Differentiation, Secretory Activity and Organelle Transfer

It was initially believed that MSCs, which are naturally endowed with multi-lineage mesodermal potential (Pittenger et al., 1999), repair injured tissues by cell-for-cell replacement driven by direct differentiation, on the model of hematopoietic stem cell transplantation. Pericyte derived human MSCs injected into cardiotoxin injured skeletal muscle do differentiate into muscle cells (Crisan et al., 2008; Park et al., 2011). Similarly, human cardiac pericytes differentiate into rare cardiomyocytes in culture and *in vivo* upon intra-myocardial injection (Chen et al., 2015).

However, the consensus is now that MSCs function in tissue repair primarily by secreting soluble factors and shedding microvesicles (Caplan, 2017). For example, promotion of angiogenesis is one of the best known mechanisms by which MSC treatment reduces scarring and promotes regeneration. Direct injection of pericyte -derived MSCs into ischemic hearts resulted in vascularization improvement in the cardiac muscle (Chen et al., 2013). In these conditions, MSCs secrete vascular endothelial growth factor (VEGF) which triggers angiogenesis (Sorrell et al., 2009). Nitric oxide synthase (NOS) secreted by MSCs can alter the ROS/RNS (reactive oxygen species/reactive nitrogen species) balance, which ultimately decreases fibrosis (Wink et al., 1999; Ferrini et al., 2002). Gnecchi et al. (2005, 2006) showed that MSC secretory activity was the main mechanism responsible for tissue protection in the ischaemic heart.

Exosomes/microvesicles shed from cell membranes are non-cellular transporters of regulatory RNAs, proteins and lipids. Extracellular vesicles (EVs) vary in shape and size with exosomes ranging between 40–150 nm in diameter, microvesicles 150–1000 nm, and apoptotic bodies 50–2000 nm. MSC-derived EVs can induce tissue progenitors to proliferate, ultimately preventing scar formation (Lai et al., 2010; Liu et al., 2018). MSC-derived exosomes have been shown to alleviate carbon tetrachloride (CCl_4_) induced liver fibrosis (Jiang et al., 2018). Although the use of MSC-derived EVs for cell therapy is promising, more research is needed to understand how these exert their regenerative benefits. In particular, EVs are themselves a heterogenous composite of vesicles, and evolving criteria for their isolation and characterization represent important guidelines for standardization in the field (Théry et al., 2018).

MSCs can also exert their healing effects by donating mitochondria to target cells. Mitochondrial transfer is an important mechanism in apoptosis prevention and metabolic damage reversion in target cells (reviewed in Paliwal et al., 2018). Perico et al. (2017) showed that human umbilical cord MSCs promote regeneration after cisplatin-induced acute kidney injury in mice. MSCs conferred to affected host cells antioxidant defense and a global metabolic switch to preserve energy supply. In this case, MSCs seem to be good candidates to alleviate the side effects of anti-cancer drugs.

## Contrasting Results in Clinical Trials: Not All MSCs Are Equal

MSCs have been used clinically for more than two decades, and over 980 registered MSC trials are listed by the FDA (www.clinicaltrials.gov). There have been more than 10,000 patients treated in a controlled clinical setting, of which 188 early trials (phase 1 or phase 2) have been completed and ten studies have advanced to phase 3 (Pittenger et al., 2019). Results have often fallen short of expectations though. In a phase III trial using MSCs (Prochymal) for treatment of steroid-refractory graft-versus-host disease (GVHD), MSC treatment showed no significant difference after 28 days compared to placebo (Martin et al., 2010). However, it was found by stratifying the study that children responded better to MSC treatment, leading to approval of Prochymal in Canada (Reicin et al., 2012). As another example, cardiopoietic primed bone-marrow derived MSCs were used to treat ischemic heart failure by the Belgium based company Celyad. Early studies suggested improvement in cardiac function. However, in subsequent trials there were no significant differences between the MSC treatment and placebo (Bartunek et al., 2013, 2016).

Depite some setbacks in clinical trials, MSC therapy has been approved in different countries. Prochymal was approved in Canada to treat acute GvHD in children, as mentioned before. In Japan, the use of MSCs was approved after the Act on the Safety of Regenerative Medicine and the Pharmaceuticals, Medical Devices and Other Therapeutic Products Act were introduced (Sipp, 2015). In 2018, the European Medicines Agency (EMA) recommended the approved Alofisel to treat Crohn’s disease (Sheridan, 2018). Overall, it appears that use of MSCs for cell therapy is becoming a reality. Nonetheless, MSC therapy in the United States has been approved by the Food and Drug Administration (FDA) in only very rare instances.

One of the highly debated aspects of MSC based medical treatments is the variable nature of the results. Many factors can influence clinical outcomes, such as MSC tissue of origin, donor gender, age, and medical history; processing of the tissue and culture conditions; freezing and thawing of the cells, and administration routes (reviewed by Galipeau and Sensébé, 2018). Furthermore, MSCs are cultured for long periods of time to obtain clinically relevant cell numbers, which results in important changes in gene expression, clonal selection, thus affecting biologic properties, including those involved in tissue regeneration.

MSCs are heterogeneous populations of cells and the diversity of existing tissue sources adds to this complexity. Bone marrow, adipose tissue and cord blood are most commonly used to obtain these cells (Gao et al., 2016), although MSCs can be obtained from virtually all vascularized organs including pancreas, skeletal muscle (Crisan et al., 2008; Corselli et al., 2012) and brain (Lojewski et al., 2015). The tissue of origin can influence the secretome of these cells (Kalinina et al., 2015). Furthermore, MSCs derived from diseased donors may show negative clinical outcomes when used for therapies (Dzhoyashvili et al., 2014). Donor age is an important factor affecting MSC efficacy. MSCs grown from neonatal tissues show a longer lifespan, higher proliferation rate and differentiation potential when compared to adult tissues (Donders et al., 2018). Furthermore, neonatal tissues are easily available, do not require invasive procedures for procurement and are ethically non-controversial.

Therapeutic MSCs should be grown in medium containing defined ingredients and no animal products. For example, the commonly used fetal calf serum is not well characterized and properties vary between batches. On the other hand, cell passaging requires the use of proteolytic enzymes which may damage the cells (Penna et al., 2015). Another aspect to take into account is oxygen concentration. High oxygen levels may compromise the therapeutic benefits of MSCs. Native MSC tissue environments range between 1 and 7% O_2_; during culture cells sense an oxygen concentration of 20%, which may cause oxidative stress affecting viability, and eventually senescence. Hypoxia increased the proliferation of MSCs when compared to standard oxygen levels used for cell culture (Zhu et al., 2016). Moreover, cells under hypoxic conditions maintain their undifferentiated status and multipotency (Basciano et al., 2011). Hypoxia also improves angiogenesis (Bader et al., 2015) and migration toward the site of injury (Vertelov et al., 2013). Another pre-conditioning tactic to improve MSC therapeutic benefits includes exposure to an inflammatory environment in the presence of IFN-γ and tumor necrosis factor alpha (TNF-α) (Krampera et al., 2006; Ren et al., 2008). Other aspects to consider are: (1) the components of culture media that may affect cell phenotype; and (2) damage caused by cryopreservation and subsequent thawing. MSCs are commonly used immediately after thawing, with no period of recovery allowed, which may impact the clinical benefit.

Contrasting results in pre-clinical studies and clinical trials using MSCs may be due to a combination of variables in organ source, donor demographics, and cell processing technical conditions. MSC treatments need to be tailored to every specific injury or disease, which may involve screening the host’s immune activation, and subsequent pre-conditioning to enhance clinical outcome (reviewed in Pittenger et al., 2019).

## Alternative Approaches to MSC Administration

Therapeutic MSCs are administered locally or systemically. Despite intense scrutiny, the fate of transplanted MSCs has not been well documented, and the study thereof complicated by the diversity of experimental and clinical settings used (autologous, allogeneic, or xenogeneic transplantation). As discussed above, injected MSC direct contribution to new tissue formation is generally minimal, with only a small fraction of xenogeneic (human) cells engrafting mouse tissues (Chen et al., 2015), and those cells not engrafted cleared from the tissue 72 h post administration (Lee et al., 2009; Gholamrezanezhad et al., 2011; von Bahr et al., 2012). It has been shown that dying transplanted MSCs engulfed by recipient macrophages release immunosuppressive soluble factors (Galleu et al., 2017), inferring that death within host tissues contributes directly to the beneficial effects of MSCs.

Conversely, autologous MSCs transplanted into the goat osteoarthritic joint persisted for several weeks and aided tissue regeneration (Murphy et al., 2003). However, this setting does not reflect the general trend, allogeneic, “off-the-shelf” MSCs being almost universally used in the clinic, principally for reasons of convenience, timely availability, and cost effectiveness.

To improve MSC driven regeneration, different approaches for collection and delivery have been envisioned. A suitable option to improve the benefit of these cells is the use of scaffolds populated by MSCs that, when engrafted, provide higher regeneration. Owing to ease of culture and broad developmental potential, MSCs have been privileged tools in tissue engineering for regenerative medicine, which uses biologicals and engineering principes to create new tissues similar to those in the human body. Tissue engineering can be used to mimic organ microenvironment for organoid culture (Fatehullah et al., 2016) and may rely on 3-dimensional printing (Poldervaart et al., 2017). The ultimate goal of tissue engineering is the replacement of the whole damaged tissue or organ, as exemplified by the engineering of tracheas (Zhang et al., 2019), hearts (Sánchez et al., 2015) and bladders (Zhe et al., 2016). Tissue engineering can also be used to improve MSC residence after administration, to which aim MSC-based scaffolds have been used, using either biodegradable or non-degradable polymers to form hydrogel matrices (Park et al., 2018), which can be supplemented with growth factors. Such matrices can be worked into desired shapes using micromolding, microfluidics, electrostatic droplet extrusion, or bioprinting (Kim et al., 2019). MSC based scaffolds systems have been used for bone and cartilage regeneration (Kim et al., 2019), as well as for the reproduction of blood vessels (Pinnock et al., 2016), cardiac tissue (Rashedi et al., 2017; Ichihara et al., 2018; Joshi et al., 2018), and skeletal muscle (Witt et al., 2017). Optimal tissue replacement efficiency relies on the physical characteristics of the scaffolds (Alakpa et al., 2017; Jeon et al., 2017; Mouser et al., 2018), as each mechanical property can modify the fate of the transplanted cells. For instance, stiff matrices can be determinant to drive MSC differentiation into chondrogenic or osteogenic cell lineages (Alakpa et al., 2016), whereas softer substrates can favor myogenic development (Gilbert et al., 2010). In addition to stiffness, dimensionality and degradability of the matrix can regulate mechanisms critical for cell differentiation (Caliari et al., 2016). It is also important to adapt the scaffold to the cell type to be used, as for instance cell size can be determinant to trigger the required mechanism (Bao et al., 2019). Overall, much work is still required before scaffold embedded MSCs can be routinely used in patients.

Alternatively, just sorted perivascular presumptive MSCs have been proposed for direct transplantation in place of cultured cells (Murray and Peault, 2015; James and Péault, 2019), the latter being susceptible to modifications hindering regenerative potentials (Zaitseva et al., 2006). In addition, expansion of MSCs selects the fastest growing clones and after enough passages, the whole cell population has become oligoclonal (Selich et al., 2016). Distinct MSC clones may also express different mesodermal differentiation potentials (Muraglia et al., 2000). Along this trend, treatment of hindlimb ischemia with freshly sorted stromal cells with high ALDH activity has yielded striking results (Capoccia et al., 2009), documenting another dramatic difference between subpopulations of MSC ancestors that may be lost after culture. Of note, a promising cell-free alternative setting alleviating problems consecutive to the use of long-term cultured cells is the transplantation of microvesicles obtained from perivascular stem cells (Xu et al., 2019).

A variation of the uncultured cell strategy relies on the administration of microfragmented adipose tissue, in which the genuine microenvironment of presumptive MSCs is maintained intact (Vezzani et al., 2019). With the tissue undisturbed by enzymatic digestion, cells sustain higher secretory activity, releasing abundant cytokines and growth factors (Vezzani et al., 2018).

In general, transplantation of uncultured cells may be ideal to improve clinical outcome, although numbers of cells obtained are lower than in culture conditions and may not be enough for proper treatment in some indications.

As a related approach, the use of autologous, intraoperatively collected cell samples for tissue regeneration has met with high interest (Coelho et al., 2012). Such cells are used extemporaneously, hence include MSC forerunners but are not *bone fide* mesenchymal stem cells, which by essence are the product of extended culture. MSC phenotype and potentials are partly shaped by culture, and it should not be assumed that the regenerative potential of a native cell population is the same as that of MSCs derived thereof. For instance, pericytes expressing the Tbx18 transcription factor readily produce MSCs in culture, but do not participate in tissue regeneration following injury (Guimarães-Camboa et al., 2017). While unmanipulated cell populations from the bone marrow, adipose tissue or other organs are clearly endowed with some tissue regenerative potential, the presence of multiple different cell types may affect this potential. It is therefore essential that the beneficial effect of freshly harvested cells be rigorously documented for each envisioned therapeutic application.

Finally, since ubiquitous presumptive MSCs have been identified in perivascular spaces that become recruited and reprogrammed in adverse disease/injury conditions, an ideal alternative to MSC administration might be the targeted pharmacologic mobilization of these cells *in situ*. To do so, we would need to understand what signals received from the diseased/inflamed environment control this transition from perivascular cells to regenerative units. For the time being, this cellular/molecular command is unknown.

## Concluding Remarks: What Future for MSCs in Medicine?

MSCs were discovered at a time when stem cell science was only nascent, and of relevance to embryonic development but to the renewal of only rare post-natal tissues. The therapeutic power of MSCs was, however, early explored, owing to the developmental potential and easy derivation of these cells from human bone marrow (Lazarus et al., 1995). Yet, the treatment with MSCs of well over 10,000 patients in almost 1000 trials has yielded variable outcomes and not yet resulted in FDA approval.

The existence of stem cells in a given tissue has been generally revealed indirectly before technical progress allowed prospective identification (Spangrude et al., 1988). Mouse embryonic stem cells, first developed from whole embryo cultures, were later identified as descending from the epiblast (Brook and Gardner, 1997). Conversely, mesenchymal stem cells were for several decades – and are still – derived principally in primary cultures of unseparated cells, since usual focus on clinical applications privileges simple and cost-effective cell production at the expense of biologic scrutiny. The more recent prospective identification and purification to homogeneity of perivascular innate MSCs has raised the possibility of using native cells for therapies, thus alleviating exposure to animal serum, oxidative stress and genetic modifications consecutive to *in vitro* culture, and allowing almost immediate use of autologous MSCs in emergency situations (Crisan et al., 2008; Corselli et al., 2012; James et al., 2012). As an alternative to costly fluorescence activated cell sorting, magnetic selection can be used to isolate regenerative human perivascular stem cells (Meyers et al., 2019b). Experiments performed so far have shown that just sorted perivascular cells are at least as potent, in terms of differentiation *in vitro* and tissue regeneration potential, as their long-term cultured MSC counterparts. Isolatable cell numbers would, however, remain an issue in many indications in which a typical therapeutic dose of MSCs is in the range of 100 million cells. For this reason, the use of non-cultured purified innate MSCs is being first explored in situations where relatively small numbers of cells can be administered locally with minimal loss, as is the case for bone repair (Tawonsawatruk et al., 2016; James et al., 2017).

Further partition, using newly identified cell surface or metabolic markers, of tissue resident MSC forerunners is currently revealing the remarkable diversity of these cells. Cell subsets committed to either osteo-, chondro-, adipo-, or fibrogenesis have been already prospectively identified. Therefore, it is expected that the combinatorial analysis of such markers will allow to circumscribe defined subsets of perivascular progenitors, as well as their MSC progeny, ideally suited for the regeneration of discrete cell lineages and tissues, notwithstanding whether this healing effect is consecutive to cell-for-cell replacement, secretion of free or microvesicle-packaged growth factors, or a combination of these actions. Importantly, better understanding of the molecular control of MSC activity and ensuing manipulation thereof should also improve the therapeutic utilization of these cells (Shen et al., 2017; Cherubini et al., 2019; Meyers et al., 2019a).

Finally, not all patients respond to MSC treatments and absent or poor responses may reflect multiple distinct factors, from MSC intrinsic quality to genetic responsiveness of the patient (Caplan, 2018). Hence the increasingly recognized importance of tuning MSCs for a given therapy, instead of using a single MSC production method for treatment of conditions as diverse as graft-versus-host disease and acute myocardial infarction. This may, for instance, involve MSC exposure to strong pro-inflammatory mediators like IL-1 prior to treating patients diagnosed with rheumatoid arthritis (Bernardo and Fibbe, 2013; discussed in Pittenger et al., 2019).

## Conclusion

In conclusion, as cell therapies for multiple different diseases are slowly becoming a reality, many stem and progenitor cell types are being considered simultaneously. Since the use of lineage-specific, tissue resident natural stem cells cannot, in most instances, be presently envisioned, transplantation of cells derived from human embryonic or post-natal pluripotent stem cells is gaining momentum, and embryonic stem cell-derived dopaminergic neurons have already been grafted with encouraging results. Will MSCs continue to be relevant therapeutic cells in years to come? MSCs have been safe and patients came unscathed through MSC treatments; the same remains to be demonstrated for ES- or iPS cell based therapies. Properties of immunomodulation and extensive growth factor release are unique, so far, to MSC-like cells. However, MSCs have long suffered from a relative lack of basic biologic investigations, and resulting empirical clinical use, the true identity of these cells being obscured by a universal retrospective derivation in culture. Recent progress succinctly collated in the present article regarding the native identity, diversity, developmental potential, secretory and immunomodulatory effects and molecular control of these cells, as well as recent insight into transcriptional modifications induced by *in vitro* culture, suggest that these cells or, ideally, their innate forerunners will be used in the future in a more controlled and analytical way, to be efficiently adapted to the patient and pathology to be treated, in a convincing example of personalized medicine.

## Author Contributions

MG-S, ZG-G, and BP conceived this manuscript. JC, MC, and AJ contributed to the writing and final review of the manuscript.

## Conflict of Interest

The authors declare that the research was conducted in the absence of any commercial or financial relationships that could be construed as a potential conflict of interest.
